# Characterization of Streptococcus pneumoniae Macrolide Resistance and Its Mechanism in Northeast China over a 20-Year Period

**DOI:** 10.1128/spectrum.00546-22

**Published:** 2022-08-08

**Authors:** Xiuzhen Zhou, Jianhua Liu, Zhijie Zhang, Bing Cui, Yanling Wang, Yue Zhang, Hailin Xu, Guixue Cheng, Yong Liu, Xiaosong Qin

**Affiliations:** a Department of Laboratory Medicine, Shengjing Hospital of China Medical University, Liaoning Clinical Research Center for Laboratory Medicine, Shenyang, China; University of Guelph

**Keywords:** macrolides, *ermB*, *Streptococcus pneumoniae*, multidrug resistance, antibiotic resistance, MDR

## Abstract

Due to the resistance of Streptococcus pneumoniae to β-lactams, macrolides, and tetracyclines, treatment alternatives have become increasingly limited worldwide. We aim to describe the characterization of erythromycin-resistant S. pneumoniae (ERSP) strains in northeastern China over a period of 20 years. A total of 1,240 ERSP strains were collected and classified into five groups based on the ages of the patients. Etest strips and Kirby-Bauer disk diffusion were performed for drug susceptibility testing. The capsule swelling test was used for capsule typing. The phenotype of drug resistance was detected by the erythromycin and clindamycin double-disk method. The *ermB*, *ermTR*, *mefA*, and *tetM* genes were detected by PCR. Among the 1,240 ERSP strains, 510 were invasive isolates, and 730 were noninvasive isolates. The results of drug susceptibility testing showed that the rates of resistance to penicillin, amoxicillin, cefotaxime, ceftriaxone, meropenem, tetracycline, trimethoprim-sulfamethoxazole, and chloramphenicol varied among the different age groups. 19F, 19A, 23F, 14, and 6B were the serotypes that were commonly found among ERSP strains. Among all strains, 99.03% (1,228/1,240) exhibited an MLSB (macrolide-lincosamide-streptogramin B) resistance phenotype, of which 1,221 strains displayed a constitutive MLSB (cMLSB) phenotype and 7 strains showed an inducible MLSB (iMLSB) phenotype. All of these strains carried the *ermB* gene. In contrast, only 0.97% of strains of M phenotypes were found to carry the *mefA* gene. Both the *ermB* and *mefA* genes were detected in 704 strains that exhibited multidrug resistance, whereas the *ermTR* gene was not detected. Furthermore, 1,185 tetracycline-resistant strains were found to carry the *tetM* gene. Macrolide antimicrobial drugs should be used cautiously for the empirical treatment of S. pneumoniae infections.

**IMPORTANCE** This study presents a retrospective analysis using 1,240 clinical erythromycin-resistant Streptococcus pneumoniae (ERSP) isolates collected in northeastern China between January 2000 and December 2019. The serotype distribution, corresponding vaccine coverage, as well as resistance phenotypes, genes, and mechanisms to macrolide and tetracycline of these isolates were systematically described, analyzed, and discussed. We hope that this study will inform clinicians in their respective regions when selecting antimicrobial agents. We also hope that this study is useful for researchers in related fields. Finally, we emphasize in this study that vaccination is the best preventive measure for S. pneumoniae infection considering its resistance to commonly used antibiotics. The determination of the S. pneumoniae serotype distribution also provides valuable empirical evidence for local health authorities when introducing appropriate vaccines in a specific area.

## INTRODUCTION

Streptococcus pneumoniae is a common respiratory pathogen in humans that colonizes the nasopharynx of healthy carriers (1, 2). It causes not only noninvasive pneumococcal diseases (non-IPDs), including community-acquired infections such as otitis media, sinusitis, and bronchitis, but also severe invasive diseases, such as sepsis, meningitis, and empyema (3). Invasive pneumococcal diseases (IPDs) can have high incidence and mortality rates in children and older adults worldwide, posing a substantial threat to public health globally. According to the World Health Organization (WHO), S. pneumoniae is the most common pneumonia pathogen, accounting for 16% of deaths in children 5 years old or younger worldwide. In developing countries, approximately 1 million children 5 years old or younger die annually from S. pneumoniae infections (4).

Surface capsular polysaccharides of S. pneumoniae are among the most important virulence factors and form the basis of S. pneumoniae serotyping. They are also the foundation of all S. pneumoniae vaccine research strategies, with over 90 different immune serotypes available. S. pneumoniae is well known for its ability to switch serotypes and acquire antibiotic resistance genes due to its ability to acquire exogenous DNA (5).

Until the 1980s, penicillin was the first-line drug used for the treatment of S. pneumoniae. This changed in the 1990s when erythromycin, azithromycin, and other macrolide antibacterial drugs began to be utilized as first-line drugs. However, as macrolide antibacterial drugs became more widely used in clinical practice, the number of erythromycin-resistant S. pneumoniae (ERSP) strains increased. According to the Asian Network for Surveillance of Resistant Pathogens (ANSORP), the rate of resistance of S. pneumoniae to β-lactams or macrolides remains high (6, 7).

Furthermore, the prevalence of multidrug-resistant (MDR) S. pneumoniae is nearly 60% (8, 9). The ability of S. pneumoniae to acquire exogenous genes has led to the resistance of S. pneumoniae to conventional antibiotics such as penicillins and macrolides, which facilitates the spread of drug-resistant strains (10). All of these have posed considerable challenges for the treatment and control of S. pneumoniae infections. The structural modification of penicillin-binding proteins (PBPs), which play an important role in cell wall synthesis, is a key mechanism of penicillin resistance. Six PBPs (PBP2b, PBP2x, and PBP1a) were among those most frequently associated with penicillin resistance in S. pneumoniae (11).

A previous study (12) described PBP changes in IPD strains in northeastern China. There are two mechanisms of macrolide resistance in S. pneumoniae. The main determinant of antibiotic resistance is the acquisition of target modifications of the *ermB* and *ermTR* genes that encode methylation enzymes (11, 13, 14). Of these two genes, the *ermB* gene mediates high levels of erythromycin resistance (MICs of ≥256 μg/mL), exhibited by the MLSB phenotype, i.e., resistance to macrolides, lincosamides, and streptogramin B. The MLSB phenotype can be further divided into constitutive MLSB (cMLSB) (erythromycin MICs of ≥256 μg/mL) and inducible MLSB (iMLSB) (erythromycin MICs of 64 to 256 μg/mL) phenotypes. The second mechanism is the acquisition of the *mefA* and *mefE* genes that encodes the active efflux pump (11, 13, 14). Isolates carrying *mef* exhibit the M phenotype (11, 13), which means that they are erythromycin resistant while being susceptible to lincomycin and streptomycin B. *mef* mediates low levels of erythromycin resistance (MICs of 1 to 16 μg/mL). In addition, the most common mechanism of tetracycline resistance in S. pneumoniae is the acquisition of one of two genes: the *tetM* gene and, less frequently, the *tetO* gene. Both of these genes encode ribosomal protection proteins (13, 14).

Resistance to erythromycin and tetracycline is usually associated with the insertion of *ermB* into the Tn*916* transposon containing *tetM*. This raises concerns about the role of tetracycline-resistant strains in the transmission of macrolide-resistant strains, in addition to the fact that the main source of *tetM* is the Tn*916* family (14).

Drug-resistant clonal strains are distributed in different countries and regions, leading to the dissemination of drug resistance phenotypes. Therefore, understanding the molecular epidemiological characteristics of S. pneumoniae in a specific region through studies can help in monitoring drug-resistant clones of S. pneumoniae and the prevalent clonal clusters in that region.

Long-term and large-scale studies characterizing ERSP strains are rare in China. Thus, to understand the characteristics of ERSP in northeastern China, we collected 1,240 nonreplicate ERSP strains during a 20-year period from January 2000 to December 2019; examined their antimicrobial susceptibility patterns, serotype distribution profiles, and erythromycin resistance phenotypes; and detected erythromycin resistance genes, including e*rmB*, *ermTR*, and *mefA*, and the tetracycline resistance gene *tetM*.

## RESULTS

### Prevalence of macrolide resistance.

A total of 1,247 S. pneumoniae strains were collected from three campuses of Shengjing Hospital, China Medical University (between January 2000 and December 2019), and 11 strains were collected from a municipal hospital (2006 to 2019). Among the 1,258 strains in total, 18 strains were erythromycin susceptible, with a susceptibility rate of 1.43% and a resistance rate of 98.57%. Among the 1,240 ERSP strains, there were 510 (41.13%) invasive S. pneumoniae strains (IPD) and 730 (58.87%) non-IPD strains. Each isolate corresponds to only one patient.

### Sources of specimens and age distribution for S. pneumoniae isolates.

Most IPD isolates (51.96%) were obtained from blood, with the remaining isolates being obtained from cerebrospinal fluid (CSF) (25.10%) and pleural fluid (19.80%). Non-IPD isolates were obtained predominantly from sputum (61.10%), with the remaining being obtained from lung lavage fluid (30.55%). Among them, sputum specimens used as a source of S. pneumoniae isolates were required to meet the following criteria: ≤10 squamous epithelial cells and ≥25 leukocytes per low-power field.

After grouping the patients by age, we ranked the IPD and non-IPD isolates according to their proportions in each age group in descending order. The proportions of IPD isolates by age group are as follows: 59.22% in the ≤2-year age group, 17.45% in the 2- to 5-year age group, 7.45% in the 5- to ≤14-year age group, 6.41% in the 14- to ≤60-year age group, and 9.41% in the >60-year age group. The proportions of non-IPD isolates by age group are as follows: 41.78% in the ≤2-year age group, 21.10% in the 2- to ≤5-year age group, 11.64% in the 5- to ≤14-year age group, 6.44% in the 14- to ≤60-year age group, and 19.04% in the >60-year age group (Table 1).

**TABLE 1:** Distribution of invasive and noninvasive S. pneumoniae strains and specimens in different age groups^*a*^

Strain type and specimen type	No. of strains or specimen types for age group					
	≤2 yrs	2 to ≤5 yrs	5 to ≤14 yrs	14 to ≤60 yrs	>60 yrs	Total
IPD	302	89	38	33	48	510
Bl	161	46	16	14	28	265
CSF	69	16	16	16	11	128
Pf	61	26	5	2	7	101
Ot	11	1	1	1	2	16
Non-IPD	305	154	85	47	139	730
Sp	178	62	40	38	128	446
BALF	98	80	41	1	3	223
Me	20	6	3	3	3	35
Se (eye)	4	3	1	2	3	13
Ot	5	3	0	3	2	13

a Bl, blood; CSF, cerebrospinal fluid; Pf, pleural fluid; Ot, other; Sp, sputum; BALF, bronchoalveolar lavage fluid; Me, middle ear fluid; Se (eye), eye secretions.

### Antibacterial susceptibility test results.

**FIG 1:**
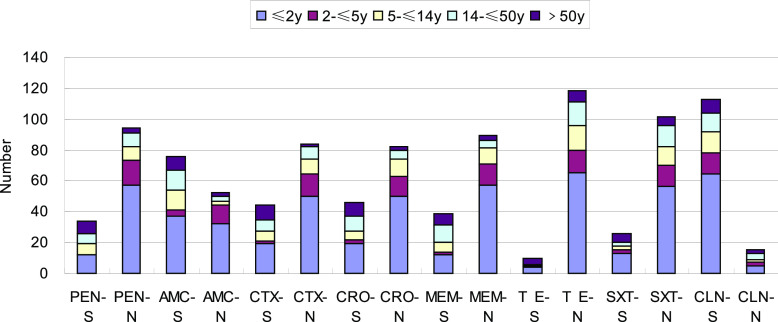
Drug susceptibility results for invasive Streptococcus pneumoniae meningitis isolates (*n* = 128). S, susceptible; N, nonsusceptible (intermediate resistance and resistance); PEN, penicillin; AMC, amoxicillin; CTX, cefotaxime; CRO, ceftriaxone; MEM, meropenem; TE, tetracycline; SXT, sulfamethoxazole; CLN, chloramphenicol.

There were 128 S. pneumoniae isolates obtained from meningitis samples. The rates of resistance to penicillin, cefotaxime (CTX), and ceftriaxone (CRO) were 82.61%, 72.46%, and 72.46%, respectively, in the ≤2-year age group and 100%, 87.50%, and 81.25%, respectively, in the 2- to ≤5-year age group. There was no difference in the drug resistance rates between the two age groups (Fig. 1).

The penicillin resistance rates in the ≤2-year and 2- to ≤5-year age groups were significantly higher than those in the other three groups (*P* < 0.01). The rate of resistance to amoxicillin in the 2- to ≤5-year age group was higher than that in the ≤2-year age group (*P* < 0.05), which was also significantly higher than those in the other three groups (*P* < 0.01). The rates of resistance to cefotaxime, ceftriaxone, and meropenem (MEM) in the ≤2-year and 2- to ≤5-year age groups were significantly higher than those in the 14- to ≤60-year and >60-year age groups (*P* < 0.01). In contrast, the rates of resistance to tetracycline and co-trimoxazole were considerably lower in the >60-year age group than in the other four groups, and the sensitivity to chloramphenicol (CLN) was higher in all age groups. The rate of resistance to sulfamethoxazole was >70% in all groups.

**FIG 2:**
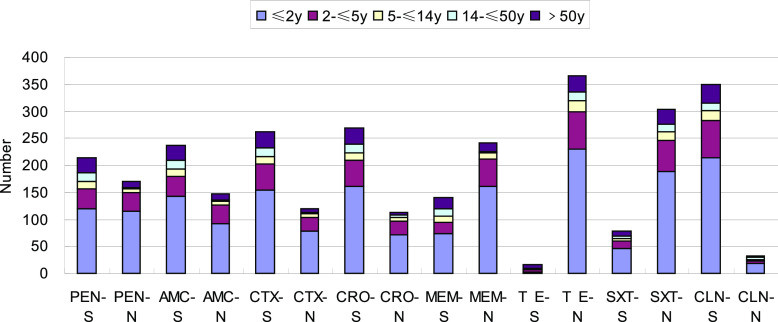
Drug susceptibility results of nonmeningitis isolates of Streptococcus pneumoniae (*n* = 382). S, susceptible; N, nonsusceptible (intermediate resistance and resistance); PEN, penicillin; AMC, amoxicillin; CTX, cefotaxime; CRO, ceftriaxone; MEM, meropenem; TE, tetracycline; SXT, sulfamethoxazole; CLN, chloramphenicol.

Figure 2 shows that the invasive non-meningitis-causing strains had lower rates of penicillin, cefotaxime, ceftriaxone, and meropenem nonsusceptibility in the 14- to ≤60-year and >60-year age groups than in the ≤2-year and 2- to ≤5-year age groups, and the rates of resistance of meningitis strains were similarly lower than those in the ≤2-year and 2- to ≤5-year age groups (*P* < 0.05).

In the noninvasive S. pneumoniae isolates, the rates of penicillin nonsusceptibility were higher in the ≤2-year, 2- to ≤5-year, and 5- to ≤14-year age groups than in the other two groups (*P* < 0.05), and the rates of susceptibility to cefotaxime, ceftriaxone, and meropenem were also higher in the ≤2-year and 2- to ≤5-year age groups (*P* < 0.05). The clinical use of penicillin for the treatment of non-meningitis-causing IPD and non-IPD isolates is based on drug susceptibility results, and higher dosage can be used if susceptibility is intermediate.

**FIG 3:**
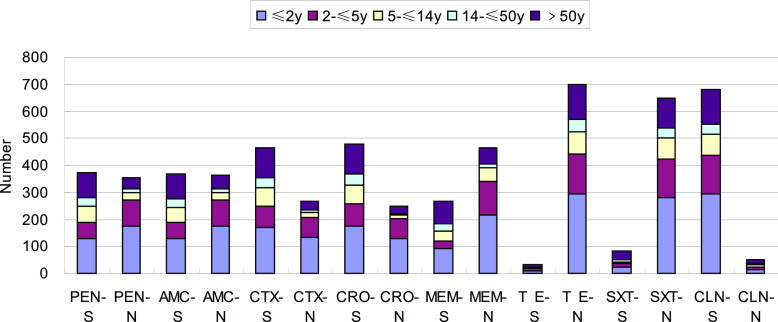
Noninvasive Streptococcus pneumoniae drug susceptibility test results (*n* = 730). S, susceptible; N, nonsusceptible (intermediate resistance and resistance); PEN, penicillin; AMC, amoxicillin; CTX, cefotaxime; CRO, ceftriaxone; MEM, meropenem; TE, tetracycline; SXT, sulfamethoxazole; CLN, chloramphenicol.

Figure 3 shows that in the ≤2-year, 2- to ≤5-year, and 5- to ≤14-year age groups, the rates of nonsusceptibility of noninvasive S. pneumoniae isolates to penicillin were lower than those in the ≤2-year and 2- to ≤5-year age groups (*P* < 0.05), and the rates of susceptibility of noninvasive S. pneumoniae isolates to cefotaxime, ceftriaxone, and meropenem were also higher in the ≤2-year and 2- to ≤5-year age groups (*P* < 0.05).

### Serotype distribution of invasive S. pneumoniae isolates in five age groups and coverage by three vaccines.

A total of 23 serotypes were detected from all invasive S. pneumoniae strains. Table 2 shows that serotype 19A was the most prevalent serotype, with 129 isolates, accounting for 25.29% of the total isolates. Serotypes 19A, 14, 19F, 23F, and 6B were predominant in the ≤2-year and 2- to ≤5-year age groups. The 58 strains of other serotypes belonged to a total of 10 serotypes: 13 strains of serotype 15; 12 strains of serotype 8; 5 strains each of serotypes 10, 11, and 22; 4 strains each of serotypes 9N, 17, and 20; and 3 strains each of serotypes 2 and 12. In all groups of S. pneumoniae polyvalent vaccines, pneumococcal conjugate vaccine 13 (PCV 13) coverage was higher than those for PCV7 and PCV10. Specifically, the ≤2-year and 2- to ≤5-year age groups were the most significant, with rates of coverage of ≥90% and a *P* value of <0.01 compared with the other three groups.

**TABLE 2:** Serotype distribution of invasive S. pneumoniae isolates in five age groups and coverage by three vaccines

Parameter	Value for age group					
	≤2 yrs	2 to ≤5 yrs	5 to ≤14 yrs	14 to ≤60 yrs	>60 yrs	Total
No. of isolates of serotype						
19F	55	17	6	3	2	83
14	59	15	6	1	4	85
23F	35	11	2	7	5	60
6B	26	6	3	3	0	38
9V	8	2	3	1	8	22
18C	4	1	0	0	0	5
4	2	1	0	0	2	5
1	3	2	1	0	1	7
5	1	0	4	0	0	5
7F	0	0	0	0	2	2
19A	84	30	3	5	7	129
6A	5	1	0	0	2	8
3	2	0	0	0	1	3
Other	18	3	10	13	14	58

Total	302	89	38	33	48	510
Coverage (%)						
PCV7	62.58	59.55	52.63	45.45	43.75	58.43
PCV10	63.91	61.80	65.79	45.45	50.00	61.18
PCV13	94.04	96.63	73.68	60.61	70.83	88.63

### Serotype distribution of noninvasive S. pneumoniae isolates in five age groups and coverage by the three vaccines.

Twenty-three serotypes were found for noninvasive S. pneumoniae isolates, with 251 isolates, accounting for 34.38% of the isolates (Table 3). Serotypes 19F, 19A, 23F, 6B, 14, and 6A were the most frequently detected serotypes in the ≤2-year and 2- to ≤5-year age groups. The rate of PCV13 coverage was higher than the rates of PCV7 and PCV10 coverage in all groups (*P* < 0.01), especially in the 2-year and 2- to ≤5-year age groups, where the rate of coverage was >90% for both groups.

**TABLE 3:** Serotype distribution of noninvasive S. pneumoniae isolates in five age groups and coverage by three vaccines

Parameter	Value for age group					
	≤2 yrs	2 to ≤5 yrs	5 to ≤14 yrs	14 to ≤60 yrs	>60 yrs	Total
No. of isolates of serotype						
19F	128	73	24	8	18	251
14	12	8	2	1	11	34
23F	33	15	15	6	20	89
6B	15	15	10	0	7	47
9V	4	1	5	4	7	21
18C	2	1	0	0	0	3
4	2	0	1	0	3	6
1	0	0	0	0	0	0
5	1	3	0	1	1	6
7F	0	0	0	2	0	2
19A	64	25	13	8	30	140
6A	17	7	3	2	4	33
3	8	1	2	0	10	21
Other	19	5	10	15	28	77

Total	305	154	85	47	139	730
Coverage (%)						
PCV7	64.26	73.38	67.06	40.43	47.48	61.78
PCV10	64.59	75.32	67.06	46.81	48.2	62.88
PCV13	93.77	96.75	88.24	68.09	79.86	89.45

### Phenotypes and genotypes of the 1,240 ERSP strains.

Table 4 shows that the cMLSB phenotype accounted for 98.47% (erythromycin MICs of ≥256 μg/mL) and that the iMLSB phenotype accounted for 0.56% (erythromycin MICs of 32 to 256 μg/mL) of the strains in this study, with *ermB* being detected in strains of both phenotypes; the M phenotype accounted for only 0.97% of the strains (erythromycin MICs of ≥1 to 8 μg/mL), in which *mefA* was detected. A total of 704 strains carrying both *mefA* and *ermB* genes were detected; drug resistance genes were detected in all strains, and no *ermTR*-positive isolates were detected.

**TABLE 4:** Drug resistance genotypes of 1,240 S. pneumoniae strains

Phenotype	No. of cases	% of cases	No. of strains resistant to:		No. of strains with genotype			
			Erythromycin	Clindamycin	*ermB*	*mefA*	*ermTR*	*ermB* + *mefA*
cMLSB	1,221	98.47	1,221	1,221	1,221	702	0	702
iMLSB	7	0.56	7	7	7	2	0	2
M	12	0.97	12	0	0	12	0	0

### Mechanisms of resistance in macrolide-resistant strains.

Among the 1,258 isolates collected, *ermB* was detected in 1,228 of all 1,240 erythromycin-resistant strains. Its rate of detection was as high as 99.03%, and the erythromycin MIC_50_ and MIC_90_ values for all age groups carrying *ermB* were ≥256 μg/mL. The rate of detection of *mefA* was 57.74%. The rate of detection of *ermB* was significantly higher than that of *mefA* (*P* < 0.01), and the rate of detection of the *ermB* and *mefA* genes was 56.77%. With the exception of IPD strains in the 14- to ≤60-year age group, the percentage of non-IPD strains carrying both the *ermB* and *mefA* genes in the other age groups was higher than that of IPD strains in the corresponding age groups (Table 5).

**TABLE 5:** Range of erythromycin MICs of strains carrying the *ermB* gene and distribution of the *ermB* and *mefA* genes by age group for IPD and non-IPD strains

Age group (yrs)	No. of isolates	MIC (μg/mL)			No. of isolates with susceptibility to erythromycin^*b*^		No. (%) of isolates with resistance determinant		
		Range	MIC_50_	MIC_90_	R	S	*ermB*	*mefA*	*ermB* + *mefA*
≤2^*a*^	304	1 to ≥256	≥256	≥256	302	2	121	2	179 (59.27)
≤2	307	1 to ≥256	≥256	≥256	305	2	100	5	200 (65.57)
2 to ≤5^*a*^	90	32 to ≥256	≥256	≥256	89	1	32	1	57 (64.05)
2 to ≤5	155	≥256	≥256	≥256	154	1	39	0	115 (74.68)
5 to ≤14^*a*^	39	≥256	≥256	≥256	38	1	23	0	15 (39.47)
5 to ≤14	86	128 to ≥256	≥256	≥256	85	1	44	0	41 (48.24)
14 to ≤60^*a*^	36	≥256	≥256	≥256	33	3	20	0	13 (39.39)
14 to ≤60	49	64 to ≥256	≥256	≥256	47	2	30	1	17 (36.17)
>60^*a*^	50	1 to ≥256	≥256	≥256	48	2	33	1	14 (29.17)
>60	142	1 to ≥256	≥256	≥256	139	3	84	2	53 (38.13)

Total	1,258				1,240	18	526	12	704 (56.77)

a Invasive Streptococcus pneumoniae.

b R, resistant; S, susceptible.

### Association of dual-gene mutations with serotype.

Dually *ermB*- and *mefA*-positive isolates were predominant in IPD and non-IPD strains in the ≤2-year age group, with strains containing both genes accounting for 25.00% (176/704) and 29.69% (209/704), respectively, followed by the 2- to ≤5-year age group, with strains containing both genes accounting for 7.95% (56/704) and 15.91% (112/704), respectively (Table 6). The *ermB* and *mefA* genes were detected in serotype 19A and 19F strains, with the exception of two 19A strains and nine 19F mucoid strains, in which only *ermB* was detected. The two serotypes together accounted for 84.09%. 19F accounted for 46.16% (325/704), 19A accounted for 37.93% (267/704), and 23F and serotype 14 each accounted for 6.68% (47/704). Serotype 6B accounted for 1.42% of the strains (10/704), and other serotypes accounted for 1.14% (8/704) (including 9V, 15, 11, and 6A). Among these strains, 25.23% of 19F, 6.44% of 19A, 34.69% of 23F, 75.56% of serotype 14 strains were isolated from IPD patients. Except for serotypes 19A and 19F, the other serotypes had dual-gene penicillin MICs of 2 to 8 μg/mL.

**TABLE 6:** Association of the dual genes *ermB* and *mefA* with serotype (*n* = 704)

Serotype	Penicillin MIC (mg/L)	No. of strains from age group									
		IPD					Non-IPD				
		≤2 yrs	2 to ≤5 yrs	5 to ≤14 yrs	14 to ≤60 yrs	>60 yrs	≤2 yrs	2 to ≤5 yrs	5 to ≤14 yrs	14 to ≤60 yrs	>60 yrs
19A	~0.12 to 32	83	30	3	4	7	64	25	13	8	30
19F	~0.25 to 16	53	16	6	4	3	126	70	23	8	16
14	~2 to 8	26	4	5	0	2	4	4	0	0	2
23F	~2 to 8	11	4	0	2	0	12	11	4	1	2
6A	4	0	0	0	0	0	0	0	0	1	0
6B	4	1	2	0	0	0	2	2	3	0	0
9V	~2 to 8	1	0	0	0	1	0	0	0	0	1
15	4	1	0	1	0	0	1	0	0	0	0
11	4	0	0	1	0	0	0	0	0	0	0

Total		176	56	16	10	13	209	112	43	18	51

### S. pneumoniae antimicrobial multidrug resistance patterns.

Table 7 shows nine MDR patterns, with total rates of MDR of 92.74% (1,150/1,240), including 94.51% (478/510) of IPD strains and 92.05% (672/730) of non-IPD strains. The penicillin G-susceptible (Pen^s^) erythromycin-resistant (Ery^r^) clindamycin-resistant (Clin^r^) azithromycin-resistant (Aq^r^) tetracycline-resistant (Te^r^) co-trimoxazole-resistant (Sxt^r^) resistance pattern was the most frequent among all MDR strains, with the *ermB* and *mefA* double mutation accounting for 46.02% (324/704) of strains with this resistance pattern, and cMLSB was the predominant phenotype. The detection of both *tetM* and *ermB* was closely related.

**TABLE 7:** S. pneumoniae antimicrobial multidrug resistance patterns^*a*^

Phenotype^*b*^ (no. of isolates)	Erythromycin MIC (μg/mL)	Resistance pattern	No. of isolates	No. of isolates with resistance determinant		
				*ermB*	*mefA*	*tetM*
Constitutive MLSB (1,221)	≥256	Pen^r^ Ery^r^ Clin^r^ Aq^r^ Te^r^	171	171	0	158
		Pen^s^ Ery^r^ Clin^r^ Aq^r^ Te^r^ Sxt^r^	659	650	324	639
		Pen^r^ Ery^r^ Clin^r^ Aq^r^ Te^r^ Sxt^r^ Mem^r^ Chl^r^	148	145	141	145
		Pen^r^ Ery^r^ Clin^r^ Aq^r^ Te^r^ Sxt^r^ Mem^r^	140	140	130	140
		Pen^r^ Ery^r^ Clin^r^ Aq^r^ Te^r^ Sxt^r^	34	34	32	31
		Pen^s^ Ery^r^ Clin^r^ Aq^r^ Te^r^ Sxt^r^	69	69	50	69

Inducible MLSB (7)	64 to ≥256	Pen^r^ Ery^r^ Clin^r^ Aq^r^ Te^r^ Sxt^r^	3	3	3	3
		Pen^s^ Ery^r^ Clin^r^ Aq^r^ Te^r^ Sxt^r^	4	4	0	0

M (12)	1 to 8	Pen^s^ Ery^r^ Aq^r^ Te^r^ Sxt^r^ Te^r^	12	0	12	0

a Ery^r^, erythromycin resistant; Sxt^r^, co-trimoxazole resistant; Te^r^, tetracycline resistant; Chl^r^, chloramphenicol resistant; Pen^s^, penicillin G susceptible; Clin^r^, clindamycin resistant; Aq^r^, azithromycin resistant; Mem^r^, meropenem resistant.

b According to the results of the double-disk diffusion method.

Among the nine MDR patterns, Pen^s^ Ery^r^ Clin^r^ Aq^r^ Te^r^ Sxt^r^ was the most common. This phenotype was dominated by cMLSB. The main resistance genes were *ermB* and *ermB* plus *mefA*.

### MLST results.

The multilocus sequence typing (MLST) results for 40 IPD strains (January 2006 to December 2008) and 23 strains (April 2016 to December 2017) are consistent. All sequence type 320 (ST320) strains presented serotype 19A, and all ST271 and ST876 strains presented serotypes 19F and 14, respectively. ST81 was associated mainly with serotype 23F. ST320 and ST271 strains carrying both the *ermB* and *mefA* genes exhibited pulsed-field gel electrophoresis (PFGE) type C and were highly correlated with Taiwan19F-14 clones (ST236, 15-16-19-15-6-20-26).

## DISCUSSION

The ERSP isolates in this study were found primarily in children 5 years old and younger. These isolates had high rates of resistance to various antibacterial drugs, particularly all macrolide antibiotics, with a rate of resistance of >95%. The most common resistance serogroup was group 19; the most common resistance phenotype was cMLSB. The resistance gene *ermB* was detected in all isolates, where more than half of the strains were found to carry both the *ermB* and *mefA* genes. *ermB* and *mefA* were highly correlated with serotypes of group 19; approximately 99.03% of the strains were MDR. The results of the MLST analysis were consistent across both periods. The most common drug-resistant clone cluster in this region was closely related to the Taiwan19F-14 clone.

In this study, the numbers of IPD and non-IPD strains were significantly higher in children (especially those who were 5 years old and younger) than in adults. In contrast, children were found to be more susceptible to IPDs and non-IPDs owing to their underdeveloped immune systems; children were found to be S. pneumoniae carriers more frequently and for longer periods than adults. Since they have more antibiotic exposure, they also face more frequent exposure to antibiotic selection pressure.

Economic development and improved medical care have led to a higher average life expectancy of the Chinese population, resulting in an aging Chinese population, with children and older adults being the groups most vulnerable to S. pneumoniae (15). The distribution of IPD specimens was primarily in the blood. The low rate of separation of CSF and pleural fluid was a result of the greater external influence and the easy autolysis of S. pneumoniae itself.

Based on the IPD and non-IPD strains shown in Fig. 1 to 3, the rate of resistance to β-lactam antibacterial drugs was considerably higher in children than in adults in this study. All strains had higher rates of resistance and nonsusceptibility to other β-lactam antibiotics. In addition, the rate of nonsusceptibility to co-trimoxazole was 70% or higher. All isolates were resistant to erythromycin and tetracycline in >95% of cases, indicating that they had little clinical utility in treating S. pneumoniae infection. Because β-lactam antibiotics and macrolides are relatively safe and less expensive for the treatment of children, they are commonly used to treat community-acquired infections in children. This chronic antibiotic pressure results in drug resistance.

According to data from the Chinese Bacterial Resistance Surveillance Study Group and the Ministry of Health National Antimicrobial Resistance Investigation Network, the rate of erythromycin resistance in S. pneumoniae in mainland China increased from 40% in 1999 to 91.9% in 2008 (16, 17), indicating a trend of a rapid increase in erythromycin resistance in S. pneumoniae. The rates of macrolide resistance in this study were notably high and did not differ by age group, with a rate of resistance to erythromycin of 98.57%, which was consistent with that found in Beijing (96.4%) (18).

Erythromycin and azithromycin are recommended for the treatment of community-acquired infections in adults and children (19). Owing to their broad-spectrum activity against both typical and atypical (particularly Mycoplasma pneumoniae) respiratory pathogens and their greater lung tissue penetration (20), macrolides are widely used for the treatment of respiratory infections. During the 20-year study period, the initial 72 strains of erythromycin-resistant S. pneumoniae accounted for 97.22% (only 2 strains were susceptible to erythromycin), implying an association between the previously high rate of total macrolide consumption and the prolonged use of macrolides such as erythromycin, clindamycin, and azithromycin (21).

The serotypes of these isolates can differ according to geographic locations, vaccine policies, and socioeconomic status. S. pneumoniae conjugate vaccines containing capsular polysaccharide antigens (PCVs) are highly effective in preventing diseases associated with S. pneumoniae and IPDs worldwide, particularly in developed countries. Serotypes change over time, according to race, age, and vaccination. Vaccines not only protect vaccinated individuals but also reduce vaccine serotype transmission, thereby providing herd protection for unvaccinated individuals in the same population (22).

However, the vaccine used must be matched to the prevalent serotype in the area. In this study, we found that the most common serotypes among 510 IPD ERSP strains (Table 2) were 19A, 14, 19F, 23F, and 6B. These findings were consistent with those observed in a previously multicenter study conducted in China (23). Among 730 non-IPD ERSP strains (Table 3), the most common serotypes were 19F, 19A, 23F, 6B, 14, and 6A. However, this finding differs from those of previous Chinese studies (18) and those of studies conducted in the United States and Europe (21, 22). PCV13 coverage was higher in all age groups for IPDs and non-IPDs than PCV7 and PCV10 coverage, particularly in the 2-year and 2- to ≤5-year age groups, where the rate of PCV13 coverage (>90%) was the highest, being significantly higher than those in the other age groups (*P* < 0.01). The protective effect of the vaccine was greater in children (5 years old and younger) than in the other age groups.

As shown in Table 4, the cMLSB phenotype was the most common phenotype among ERSP strains in northeastern China (98.47%), with only 7 (0.56%) strains with the iMLSB phenotype and 12 (0.97%) strains with the M phenotype being detected in this study. This is consistent with the findings of previous research in China (18). Conversely, other countries, such as the United States and the United Kingdom, have higher prevalences of the M phenotype (24, 25). The overall rate of detection of *ermB* was 99.03%, indicating that *ermB* is primarily responsible for macrolide resistance in the northeastern region. In addition, the total rate of detection of *mefA* was 57.74%, whereas that of the M phenotype was only 0.97%. The results of this study are consistent with those of previous and recent studies in China, with a predominance of the cMLSB phenotype and a few strains of the M phenotype (18, 24, 26). *ermTR* was not detected in this study, suggesting that macrolide resistance is not associated with it in northeastern China.

The phenotypic and genotypic characteristics of ERSP strains vary geographically. *ermB* is the most common genotype globally and also the most common mechanism of erythromycin resistance in Asia, including mainland China, Taiwan, Sri Lanka, Japan, and South Korea (6, 7). Those findings are consistent with the findings for S. pneumoniae in northeastern China in this study. In contrast, *mefA* is more common in Hong Kong, China, Singapore, Thailand, and Malaysia (6, 7). Moreover, until the introduction of PCV7 in the United States in 2000, *mefA*-mediated resistance was the most common mechanism of macrolide resistance (24). In Europe, *mefA* was found to be more common in the United Kingdom (70.8%), Greece (66.2%), Australia (59.5%), Finland (55.4%), and Germany (53.2%) (25); furthermore, *ermB* was common in Belgium (91.5%), France (90%), Spain (88.3%), Serbia (82.4%), Poland (80.8%), and Italy (55.8%) (27).

The rate of resistance to tetracycline was also high in S. pneumoniae strains in mainland China in the ANSORP study, which may be associated with the misuse of tetracycline in agriculture and edible animals (18). In conjunction with previous research, the current study established *tetM* as a mechanism of tetracycline resistance in S. pneumoniae (18). The rate of tetracycline resistance in this study was 95.56%, whereas *tetM* was found in all tetracycline-resistant strains and was substantially associated with *ermB*. This association appears to be caused by the insertion of *ermB* into a complex transposon of the Tn*916* family containing *tetM* (28, 29).

Differences in macrolide prevalence and resistance genotypes can be attributed to the transmission of resistant clones and different patterns of macrolide use, resulting in variation in the resistance genotype (18). In our study, 56.77% (704/1,240) of the strains carried both the *ermB* and *mefA* genes, consistent with the findings in previous reports from China (18). The MIC_50_ and MIC_90_ of erythromycin in all age groups carrying both the *ermB* and *mefA* genes were ≥256 μg/mL, and all penicillin-resistant strains were found to carry both genes. The occurrence of dually *ermB*- and *mefA*-positive strains has been observed worldwide. In the present study, the serotypes of most (84.09%) of the isolates carrying both the *ermB* and *mefA* genes were 19F and 19A. These findings suggest that the prevalence and distribution of serotypes 19F and 19A in northeastern China contribute to the high incidence of macrolide-resistant S. pneumoniae in the region. Strains of serotypes 19F and 19A with both the *ermB* and *mefA* genes were found in 77.27% (136/176) of children in the ≤2-year age group with IPDs, 82.14% (46/56) in the 2- to ≤5-year age group with IPDs, 90.91% (190/209) in the ≤2-year age group with non-IPDs, and 84.82% (95/112) in the 2- to ≤5-year age group with non-IPDs. Thus, children under 5 years of age are a group with a high prevalence of macrolide resistance in northeastern China.

The development of resistance to three or more different classes of antibiotics is described as MDR. More than 30% of S. pneumoniae strains reportedly exhibit MDR globally (30). In a 2004–2005 study conducted in 15 European countries, 15.8% of S. pneumoniae strains exhibited MDR, with 40.8% in France and 42.9% in Greece (31), whereas MDR is more common in Asia. According to the ANSORP surveillance study, the total rates of MDR in Asia were 26.8% from 2000 to 2001 (6) and 59.3% from 2008 to 2009, with mainland China having the highest percentage, at 83% (32). In this study, the rate of MDR of IPD strains was 94.51% (478/510), and that of non-IPD strains was 92.05% (672/730). MDR S. pneumoniae infections are important because they can resist standard treatment with β-lactams and macrolides.

S. pneumoniae has a built-in ability for genetic transformation (33). Its ability for horizontal gene transfer enables the organism to adapt to environmental changes such as antibiotic pressure. Indeed, one of the reasons for the emergence of MDR may be the greater abilities of S. pneumoniae.

The MLST database facilitates global data sharing and data exchange between laboratories. Thus, clonal complex 271 (CC271) appeared after the introduction of PCV7 in the United States and the simultaneous expression of *ermB* and *mef* genes (34). Our previous analyses of 63 IPD strains by MLST (15, 16) revealed that all ST320 strains were of serotype 19A, whereas all ST271 and ST876 strains were of serotypes 19F and 14, respectively, and ST320 and ST271 isolates carrying both *ermB* and *mefA* exhibited type C and were highly correlated with the Taiwan19F-14 clone (ST236, 15-16-19-15-6-20-26). Both ST320 and ST271 strains are present in CC271, with the Taiwan19F-14 clone belonging to CC271. It carries *ermB*, *mefA*, and *tetM* and is resistant to penicillin, erythromycin, and tetracycline. Our data confirm that the transmission of these Taiwan19F-14 clones is the main reason for the simultaneous presence of *ermB* and *mefA* in S. pneumoniae isolates from this region (34).

In this study, 85 mucus-type S. pneumoniae strains were isolated, 3 of which were susceptible to erythromycin, and 17 serotypes were detected among 82 erythromycin-resistant strains. Of note, only the *ermB* gene was detected in all mucus-type ERSP strains, but no *mefA* gene was detected. This has not been reported in previous studies, and the cause of the lack of detection of *mefA* in the 11 mucus group 19 S. pneumoniae strains requires further investigation.

The long period of 20 years, the large number of strains, and the stratified analysis of invasive and noninvasive strains are all advantages of this study. However, it also has two limitations. First, it is a single-center research study and may not represent the prevalent trend across China. Second, due to limited financial resources, we were not able to perform MLST analyses on all strains.

In summary, the rates of resistance to macrolides and tetracycline were notably high in northeastern China, attributable to resistance mechanisms such as the *ermB* and *tetM* genes that encode methylation enzymes. Therefore, macrolides and tetracycline have little clinical value in the treatment of S. pneumoniae. The most common resistant clonal serotypes were 19A-ST320, 19F-ST271, and 14-ST876. The Taiwan19F-14 clone was the primary source of MDR with both the *ermB* and *mefA* genes. Moreover, Taiwan19F-14 clone transfer was the route of transmission of S. pneumoniae macrolide and tetracycline resistance in this region.

## MATERIALS AND METHODS

### Isolation and identification of strains.

Strains were collected from three campuses of Shengjing Hospital of China Medical University, a large regional hospital with over 6,000 beds and 4.7 million outpatient visits per year. For the accurate identification of species, all strains were identified either by using standard colonial morphology, Gram stain morphology, an optochin susceptibility test, and a bile lysis test or by using a Vitek-2 compact GP identification card and Vitek mass spectrometry. All isolates were stored at −80°C in skimmed milk medium until further analysis. S. pneumoniae standard strain ATCC 49619 was purchased from the China Medical Strain Center in Beijing.

This study was approved by the ethics committee of Shengjing Hospital. All procedures were performed according to the Declaration of Helsinki (2008 revision).

### Antibacterial susceptibility test.

Among the 1,240 ERSP strains, the MICs of penicillin, erythromycin, clindamycin, tetracycline, and azithromycin were determined using Etest strips (AB Biodisk, Solna, Sweden). The Vitek 2 compact system (bioMérieux, Marcy l’Etoile, France) was used to determine the MICs of amoxicillin, cefotaxime (CTX), ceftriaxone (CRO), meropenem (MEM), sulfamethoxazole (SXT), and chloramphenicol (CLN). Breakpoints were determined using Clinical and Laboratory Standards Institute (CLSI) revised criteria from 2019 (35). MDR strains appear to be resistant to three or more different classes of antibiotics. S. pneumoniae ATCC 49619 was used as a quality control strain.

### Serotypes.

The isolates were serotyped using type-specific antisera in the Quellung test (Statens Serum Institut, Copenhagen, Denmark). Phase-contrast microscopy was used for serotyping, as previously described (36). The percentages of isolates expressing the serotypes included in the vaccine were used to calculate the rates of pneumococcal conjugate vaccine PCV7, PCV10, and PCV13 coverage.

### Erythromycin resistance phenotype detection by the double-disk synergy method.

The drug resistance phenotypes of all 1,240 ERSP strains were determined by the Kirby-Bauer disk diffusion method using erythromycin (15 μg) and clindamycin (2 μg) paper disks, as described by the CLSI (35). When both disks are without an inhibition zone, the phenotype is considered constitutive resistance to macrolides, lincosamides, and streptogramin B (cMLSB). When the erythromycin disk is without an inhibition zone while the clindamycin disk shows a defective inhibition zone (D style), the phenotype is defined as induced resistance to macrolides, lincosamides, and streptogramin B (iMLSB). If the erythromycin disk shows drug resistance while the clindamycin disk shows that the strain is susceptible to clindamycin, the strain is defined as belonging to the M phenotype.

### Genomic DNA extraction.

Chromosomal DNA was extracted from S. pneumoniae cells incubated overnight in Columbia blood agar flat dishes in a 5% CO_2_ environment according to the manufacturer’s instructions. The TIANamp bacterial DNA kit (Tiangen Biotech, Beijing, China) was used to extract bacterial genomic DNA. The extracted DNA was stored at −20°C until use.

### Detection of the erythromycin resistance genes *ermB*, *ermTR*, and *mefA* and the tetracycline resistance gene *tetM* by PCR amplification.

The following four sets of primers were used for gene amplification (5′ to 3′): *ermB* forward (F) primer 5′-GAA AAG GTA CTC AAC CAA ATA-3′ and reverse (R) primer 5′-AGT AAC GGT ACT TAA ATT GTT TAC-3′ (37), *mefA* F primer 5′-AGT ATC ATT AAT CAC TAG TGC-3′ and R primer 5′-TTC TTC TGG TAC TAA AAG TTG-3′ (37), *ermTR* F primer 5′-AGA AGG TTA TAA TGA AAC AGA A-3′ (38) and R primer 5′-GGC ATG ACA TAA ACC TTC AT-3′, and *tetM* F primer 5′-GAA CTC GAA CAA GAG GAA AGC-3′ (39) and R primer 5′-ATG GAA GCC CAG AAA GGA T-3′.

PCR was run using a 50-μL reaction mixture containing ~40 ng of the DNA template, 0.2 μM each primer, 2.5 mM each deoxynucleoside triphosphate (dNTP), 1.5 U *Taq* DNA polymerase, and 1× PCR buffer. An initial denaturation step at 94°C for 5 min was followed by 35 amplification cycles of 94°C for 40 s, 60°C for 40 s, and 72°C for 50 s. A final extension step was performed at 72°C for 7 min. The annealing temperatures for *ermB*, *mefA*, *ermTR*, and *tetM* were 56°C, 52°C, 57°C, and 58°C, respectively. The expected PCR product sizes were 639 bp for *ermB*, 348 bp for *mefA*, 530 bp for *ermTR*, and 740 bp for *tetM*.

Electrophoresis was performed on a 1.5% agarose gel with 0.5 μL PCR products and 1.0 μL 6× loading buffer for 45 min (Tanon Eps300). The voltage and current were set to 150 V and 100 mA, respectively. Images were obtained and recorded using a PAC3000 gel photograph system (Bio-Rad, USA).

### Detection of drug resistance genes.

The macrolide resistance genes *ermB*, *ermTR*, and *mefA* and the tetracycline resistance gene *tetM* were detected by PCR amplification using the four primer pairs and the experimental conditions described above.

### Multilocus sequence typing analysis.

Multilocus sequence typing (MLST) analysis was performed on 40 strains isolated from children less than 5 years old between January 2006 and December 2008 and 23 invasive S. pneumoniae strains isolated from April 2016 to October 2017 in two multiple-center research studies described previously (15, 40).

### Statistical analysis.

Antibiotic susceptibility analysis was performed using WHONET 5.6 software (WHO, Geneva, Switzerland). The χ^2^ test was used to compare proportions using SPSS19.0 software. A *P* value of <0.05 was considered statistically significant.
